# Pure menstrual migraine with sensory aura: a case report

**DOI:** 10.1007/s10194-012-0450-9

**Published:** 2012-04-24

**Authors:** Jiann-Jy Chen, Yung-Chu Hsu, Dem-Lion Chen

**Affiliations:** 1 Department of Medical Imaging, Taipei Medical University, Shuang Ho Hospital, New Taipei, Taiwan; 2 Department of Neurology, Ditmanson Medical Foundation Chia-Yi Christian Hospital, No. 539, Chung-Shao Road, Chia-Yi City, 60002 Taiwan; 3 G-Home Clinic, Kaohsiung, Taiwan

**Keywords:** Menstrual migraine, Migraine aura, Sensory aura, Peri-menopausal

## Abstract

Hormonal changes related to the menstrual cycle have a great impact on migraines in women. Menstrual migraine attacks are almost invariably without aura. Categorizing migraines into menstrual or non-menstrual types is one way to stratify migraines without aura according to the appendix criteria of the International Classification of Headache Disorders. We report a peri-menopausal woman whose sensory aura exclusively heralded menstrual migraine. A 51-year-old woman had suffered from monthly episodic headaches since the age of 46. Before a headache, and within 1 h on the first day of her menstruation, she always experienced numbness in her entire left upper limb. After the sensory aura, migrainous headaches occurred with nausea and photophobia. In the postmenopausal period, she no longer had sensory aura, and her headache pattern changed and became less severe. Her physical and neurologic exams as well as electroencephalography, brain magnetic resonance imaging, and conventional angiography were all normal. She fulfilled the diagnosis of pure menstrual migraine with typical sensory aura. To our knowledge, this is the first formal case report of pure menstrual migraine with aura.

## Introduction

Hormonal changes, especially related to the menstrual cycle, have a great impact on the occurrence of migraines in women. The use of menstrual or non-menstrual migraines has been one way to stratify migraines without aura according to the appendix criteria of the International Classification of Headache Disorders, second version (ICHD-2) [1]. Menstrual migraines (MM) are further classified into pure menstrual migraines (PMM) and menstrually related migraines (MRM), which affect 10–14 % and over 50 % of female migraineurs, respectively [2]. Women having migraines without aura encounter MM more frequently than those having migraines with aura [3]. Although a MRM sufferer may experience a migraine with aura outside the menstrual period, MM attacks almost invariably occur without aura [4]. However, we diagnosed a female patient whose PMM with aura exclusively occurred at the onset of menstruation in the peri-menopausal years. From our literature review, we believe this is the first formal report of PMM with aura.

## Case report

A 51-year-old woman had been healthy and denied head trauma, coffee or alcohol consumption, and contraceptive drug use history. She did not have postpartum headache in the past. However, she had suffered from monthly episodic moderate headaches since age 46 and they always occurred on the first menstrual day. Premonitory symptoms included 1 week of abdominal fullness and poor appetite.

She always experienced sensory aura as hypoesthesia at her left hand and gradually extended to whole upper limb within 20–30 min. It disappeared also from her hand and then completely remission in another 20–30 min. The whole process did not involve face, trunk, or other limbs. She did not have visual or language auras. After remission of the sensory aura, headache ensued immediately and was described as holocranial, throbbing, and outward expanding in nature. Associated symptoms included photophobia, nausea, and occasional vomiting without phonophobia. Physical activities exacerbated the pain; therefore she was confined to bed. The headache usually lasted for 6 h despite taking 500 mg acetaminophen from local clinic. After the headache, euphoria would occur during the two menstrual days that followed. She denied having isolated aura without headache through her life.

She began menopause half a year ago, and did not receive hormone replacement therapy. Her headache pattern then changed, with the headache still occurring regularly at intervals of once per month, but each attack lasted only for one-half to 2 h. She no longer experienced aura or premonitory symptoms. The headache remained holocranial, but became a dull pain in nature. Associated symptoms included photophobia rather than phonophobia, nausea or vomiting. Physical activities did not exacerbate her pain, so she could work or ambulate as usual.

Due to the atypical presentation of MM, she came to visit us and further evaluations were arranged for her. General physical and neurological exams were normal. Electroencephalography, brain magnetic resonance imaging, and conventional angiography showed no abnormality. Blood tests, including anti-nuclear antibodies, were all within normal limits. She fulfilled the diagnostic criteria of PMM with typical sensory aura. As the current headache simply recurred each month and did not affect her much, 500 mg acetaminophen was still recommended for acute symptomatic relief.

## Discussion

We reported a peri-menopausal woman whose late-onset PMM was associated exactly with sensory aura. Previous epidemiologic study of migraine with typical aura showed 99 % in the form of visual aura [5]. However, our patient had uniqueness of pure sensory aura. In addition, she also had a longer period of premonitory symptoms and post-dromal euphoria. Although MM is usually more severe and resistant to treatment [6], our patient’s headache had a relatively good response to acetaminophen 500 mg. So she never received triptan for abortive treatment.

MM tends to be associated with migraine without aura. In a pilot study of 55 migraineurs, MacGregor et al. [7] found that none of the PMM was migraine with aura. She advocated new appendix criteria for the ICHD-2 [2], which classified female migraine without aura into PMM, MRM, and non-MM. However, some studies showed opposing results. In a retrospective clinic-based study [3], migraine with aura attack only in the menstrual period had a prevalence similar to migraine without aura (4 % vs. 3 %), but Granella et al. attributed this result to selection bias from clinics because migraine with aura drove patients to visit clinics. Another questionnaire-based study showed menstruation may be a trigger of migraine with aura, but did not clearly describe PMM or MRM [8]. In conclusion, several epidemiological studies mention migraine with aura occurring menstrually, but clinical details are usually lacking and one cannot be sure if these papers refer to PMM or instead to MRM.

The pathophysiology of MM is associated with falling estrogen levels in the late luteal/early follicular phase of the menstrual cycle [4]. Furthermore, MM tends to improve with age, especially following menopause [9]. However, in the peri-menopause years, the disordered and often unpredictable hormonal fluctuations may worsen previous migraines, or even contribute to the new onset or rebound of subsided migraines [2]. In our case, although the patient’s pre-menopause life was uneventful, estrogen concentrations fluctuated chaotically during the peri-menopausal years, exposing her to migraine with aura during the onset of each menstruation. It seems convincing that the decreased and less fluctuated estrogen level after menopause had abated the typical aura and premonitory symptom [7], and even changed the headache pattern after menopause.

Normally, the relationship of migraine attacks with menstrual cycle is ascertained by a headache diary. Since our patient had regular occurrence of attacks on the first day of menses only, a diagnosis of PMM by history can be acceptable. The lack of a headache diary, however, should be underlined as a limit of our case report.

In conclusion, we reported an unusual case of a female patient who had PMM with a distinct and regular aura. Her aura ceased and the migraine features became less prominent after menopause.

## Abbreviations

MM: Menstrual migraine
PMM: Pure menstrual migraine
MRM: Menstrually related migraine
ICHD-2: International Classification of Headache Disorders, second version
